# Aging induces a step-like change in the motor ability structure of athletes

**DOI:** 10.18632/aging.102126

**Published:** 2019-07-30

**Authors:** Siddhartha Bikram Panday, Prabhat Pathak, Jeongin Moon, Jooeun Ahn

**Affiliations:** 1 Department of Physical Education, Seoul National University, Seoul 08826, Republic of Korea; 2 Department of Sports and Leisure Studies, Keimyung University, Daegu 42601, Republic of Korea; 3 Institute of Sport Science, Seoul National University, Seoul 08826, Republic of Korea

**Keywords:** motor ability structure, principal component analysis, masters athletes, strength, endurance

## Abstract

Many studies have investigated how aging decreases human strength and endurance. However, understanding the effect of aging on human motor ability requires more than knowledge of the separate temporal profile of individual motor function because the structure of human motor ability is multi-dimensional. We address the effect of aging on the multi-dimensional structure of human motor ability by investigating the performance records of athletes in track events across various age groups. We collected the performance records of 446 top-level decathletes whose ages ranged from 20 to 74, and performed a principal component analysis of the records in 100m, 1500m, and 400m races, which require strength, endurance, and the mixture of both, respectively. Our analysis shows that aging results in a substantial and sudden change in the motor ability structure, contrasting sharply with the gradual decrease in performance in each track event. The rapid structural change develops around the age of 50, which is much earlier than the “breakpoint” of 70 years suggested in multiple previous studies. Our findings indicate that the structural change in motor ability can significantly precede the failure in the overall motor performance.

## INTRODUCTION

Maintaining motor performance at an appropriate level is a common desire among humans, which cannot be fulfilled owing to numerous factors, such as disease, injury, and aging. Among these factors, aging is the one currently affecting all humans. Strihler characterized aging as the structural and functional deterioration of the overall neuromuscular system, and the corresponding degradation of the physio-motor abilities [1]. Although the underlying mechanism of aging has not been fully clarified despite extensive studies in various fields, most studies in the literature agree that aging-related complications are not simply the result of chronological age; a mixture of various factors induces aging, and ultimately decreases the overall motor performance [2–4]. The fact that aging is affected by numerous factors including chronological age, genetics, environment and lifestyle has hampered systematic and quantitative research on the effect of aging on human motor ability; controlled experiments with human participants have been challenging.

Among the factors affecting aging apart from the chronological age, training is a flagship example. The negative effects of aging are unavoidable for all individuals, but they are more prominent among non-athletes. Strength parameters like number of motor units, rate of force development, and peak power of elite athletes are better than those of healthy age-matched controls [5, 6]. The decline in the maximum rate of oxygen consumption due to aging also depends on the amount of training [7, 8], and the aerobic capacity of highly trained octogenarians is substantially higher than that of their untrained counterparts [9].

In attempts to focus on the pure effect of age, several studies investigated the performance of masters athletes who underwent a similar level of training, and showed that parameters, such as muscle mass, strength, speed, power, and endurance, all deteriorate with increasing age, even in master athletes [4–6, 10, 11]. Particular attention has been paid to the two key parameters of human motor ability: strength and endurance. Some studies claimed that a decrease in strength precedes a decrease in endurance [12, 13], whereas others claimed the opposite [10, 14, 15]. In addition, Rittweger et al. suggested a balanced decline in both [11].

However, human motor ability is multi-dimensional, and therefore, understanding the effect of aging on human motor ability requires more than simple knowledge of the temporal profile of each motor function. Knowledge of how aging affects the structural aspects of motor ability is needed as well. In this study, we address the effect of aging on human motor ability structure by extracting the principal components (PCs) from the 100m, 400m, and 1500m run records of 446 top-level male decathletes whose ages ranged from 20 to 74. It is generally accepted that the record in 100m run represents sprinting ability, i.e., strength and power, whereas the performance in 1500m run represents endurance [16–18]. An athlete’s performance in a 400m run indicates the mixed motor abilities of strength and endurance [16, 17, 19]. The results of our principal component analysis (PCA) clarifies that 1) the relative dominance of strength and endurance in each PC, or multi-dimensional structure of motor ability of masters decathletes changes substantially due to age, and 2) the change mostly develops around 50, which significantly precedes the “breakpoint” suggested in previous studies [20, 21].

## RESULTS

We categorized the 446 record holders into the following nine age groups: 20~34, 35~39, 40~44, 45~49, 50~54, 55~59, 60~64, 65~69, and 70~74 years. The sample size, the average speed, and the standard deviation of each age group and each track event are summarized in Table 1.

**Table 1:** Sample size and running performance of different age groups.

		**Average Speed (m/s)**					
		**100m**		**400m**		**1500m**	
**Age**	**Sample Size**	**Mean (SD)**	**Decline (%)**	**Mean (SD)**	**Decline (%)**	**Mean (SD)**	**Decline (%)**
20~34	109	9.13 (0.18)	*Baseline*	8.12 (0.16)	*Baseline*	5.44 (0.18)	*Baseline*
35~39	43	8.75 (0.23)	−4.16	7.67 (0.18)	−5.65	5.15 (0.26)	−5.19
40~44	34	8.32 (0.29)	−8.86	7.27 (0.22)	−10.54	4.86 (0.30)	−10.61
45~49	58	8.11 (0.28)	−11.17	7.05 (0.24)	−13.18	4.80 (0.31)	−11.72
50~54	52	7.85 (0.23)	−13.97	6.76 (0.22)	−16.83	4.59 (0.35)	−15.63
55~59	40	7.57 (0.23)	−17.04	6.42 (0.23)	−20.99	4.34 (0.32)	−20.25
60~64	44	7.39 (0.24)	−19.00	6.12 (0.32)	−24.73	4.04 (0.37)	−25.69
65~69	29	7.11 (0.19)	−22.09	5.83 (0.39)	−28.23	3.86 (0.40)	−29.00
70~74	37	6.78 (0.25)	−25.69	5.43 (0.28)	−33.13	3.52 (0.33)	−35.26

Note. Decline in the average speed is calculated as the percentage change with respect to the performance of 20~34 group.

### Changes in the patterns of factor loadings

**Figure 1:**
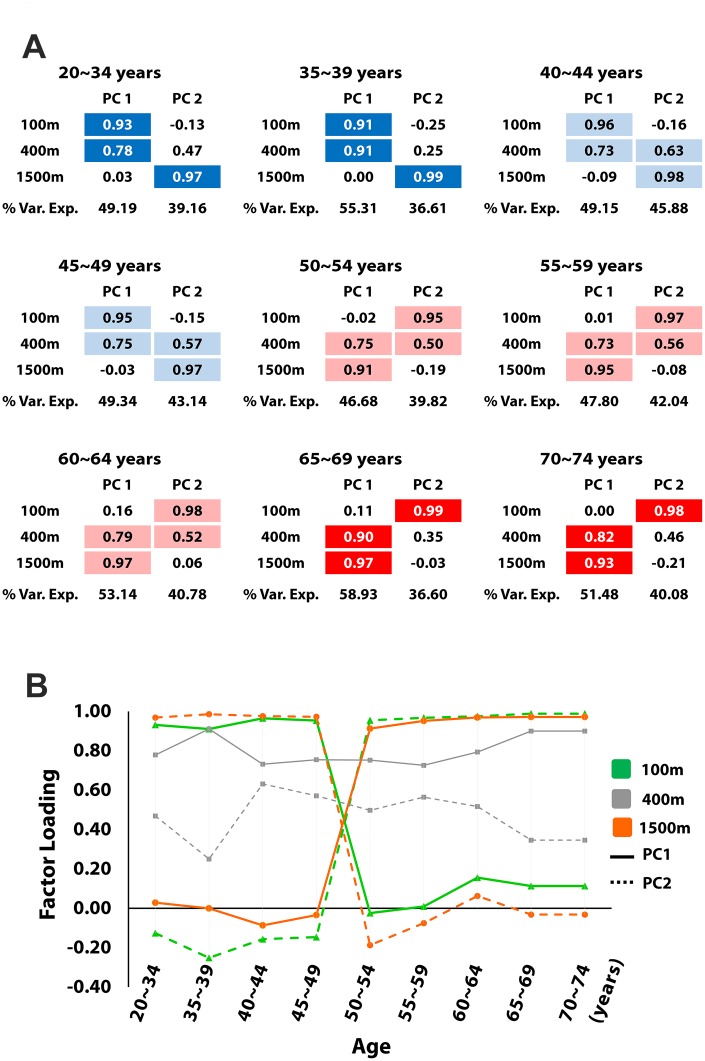
**The change in loading patterns.** (**A**) shows the factor loadings for the first and the second principal components (PCs) and proportions of variance explained by the PCs. Colors are assigned to loadings ≥ 0.5. Four different colors indicate four categories classified according to the loading pattern. (**B**) shows how each factor loading changes as the age increases. Flipping occurs around the age of 50.

The factor loadings (correlation coefficients) for the extracted PCs, and the proportions of variance explained by the PCs are shown in Figure 1. The loading pattern is visualized by highlighting significant factor loadings ≥ 0.5. According to the loading pattern, the nine age groups were classified into four distinct categories. The loading pattern of the 20~34 and the 35~39 age groups shows high contributions of the 100m and the 400m runs to the formation of the first PC, and a high contribution of the 1500m run to the second PC. A similar, but slightly different loading pattern is observed in the 40~44 and the 45~49 age groups. The dominance of performance in the 100m and the 400m runs remains in the first PC, but the performance in the 400m run newly shows a significant contribution to the second PC. *A substantial difference is observed between the 45~49 and the 50~54 age groups; the loading pattern is flipped as the average age increases only by 5 years.* The loading pattern of the 50~54, the 55~59, and the 60~64 age groups shows high contributions of the 1500m and the 400m runs to the first PC; and a high contribution of the 100m run and a significant contribution of the 400m run to the second PC. This loading pattern changes only slightly in the two oldest groups; the loading pattern of the 65~69, and the 70~74 age groups shows high contributions of the 1500m and the 400m runs to the first PC, and a high contribution of the 100m run to the second PC.

**Figure 2:**
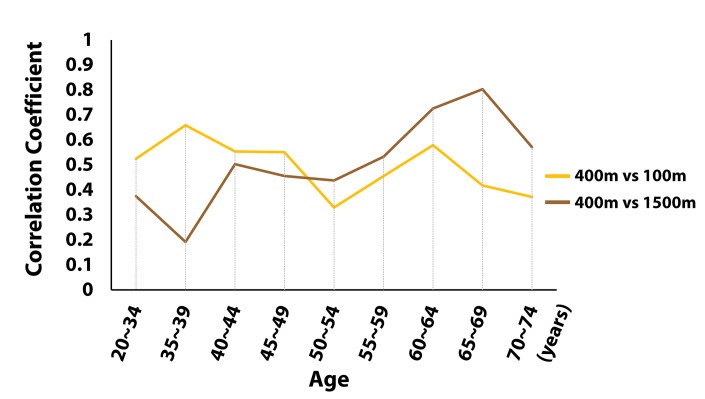
**The change in correlation coefficients.** The age dependent changes of the correlation coefficient between the speed in 100m run and the speed in 400m run; and the correlation coefficient between the speed in 400m run and the speed in 1500m run are shown. The two correlation coefficients cross around the age of 50.

The clear contrast between the loading patterns of the 70~74 and the 20~34 age groups shows that aging significantly affects the relative dominance of each factor loading. However, this substantial change does not develop gradually with the increase in age. The structural change is minimal between any pair of neighboring age groups, except between the 45~49 and the 50~54 age groups, where the flipping of the loading patterns occurs. Figure 2 additionally shows how the correlation between the performance in 100m run and 400m run and the correlation between the performance in 400m run and 1500m run change as the age increases. The correlation coefficients also cross at the age of 50, when the flipping of the loading pattern occurs.

### Rapid changes in the directions of PCs

**Figure 3:**
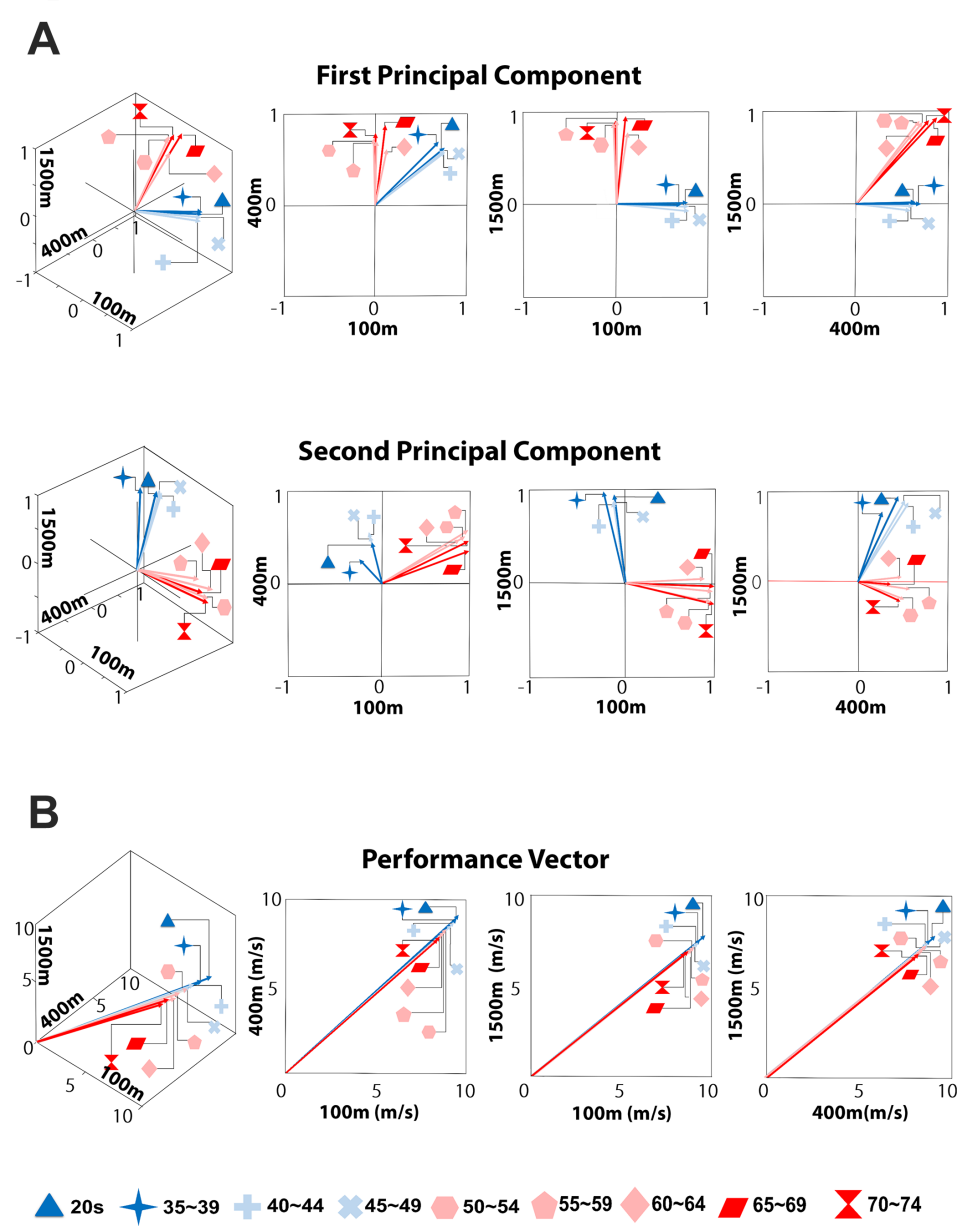
**The change in principal components (PCs) and performance.** (**A**) Abrupt changes in the directions of the PCs. Each vector denotes the first or the second PC. Sudden changes in the directions of both the first and the second PCs occur around the age of 50. (**B**) Gradual decrease in performance. The coordinate of the end point of each vector consists of the mean values of average speed in three track events. Aging gradually diminishes the magnitude of the vector, but hardly changes its direction.

The step-like change in the loading pattern is additionally visualized in Figure 3A, which shows the directions of the PCs, or the eigenvectors of the covariance matrix of the performance records. The directions of the eigenvectors of age groups below 50 years are distinct from those above 50, whereas the directions of the eigenvectors remain relatively similar before or after the age of 50 years. By contrast, the motor performance itself shows gradual decrease. The vectors whose components represent the mean values of average speed in the 100m, 400m, and 1500m races across all age groups are presented in Figure 3B. The vector magnitude decreases gradually with increases in age, whereas no significant or rapid change in the direction of the vector is observed.

### Gradual decreases in performance

**Figure 4:**
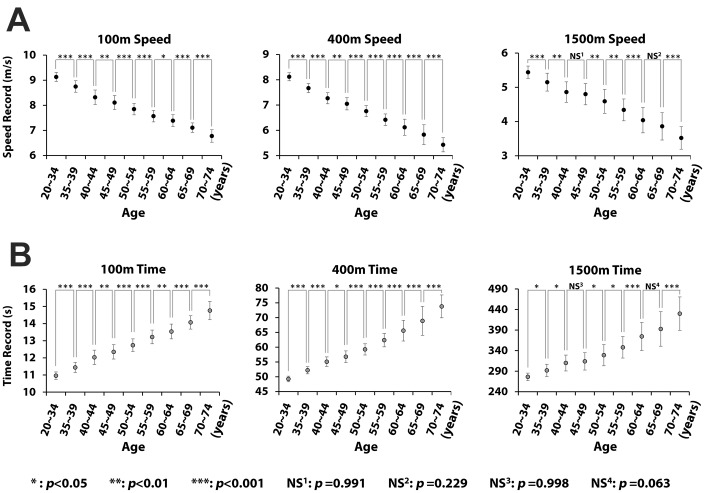
**Statistically significant decrease in performance with increase in age.** (**A**) and (**B**) show 100m, 400m, and 1500m run performance in speed and record times across nine age groups. The circle and the bar denote the mean and the mean ± standard deviation. Analysis of variance (ANOVA) demonstrates significant differences between all pairs of neighboring age groups in all three events, except only two pairs in the 1500m run.

**Figure 5:**
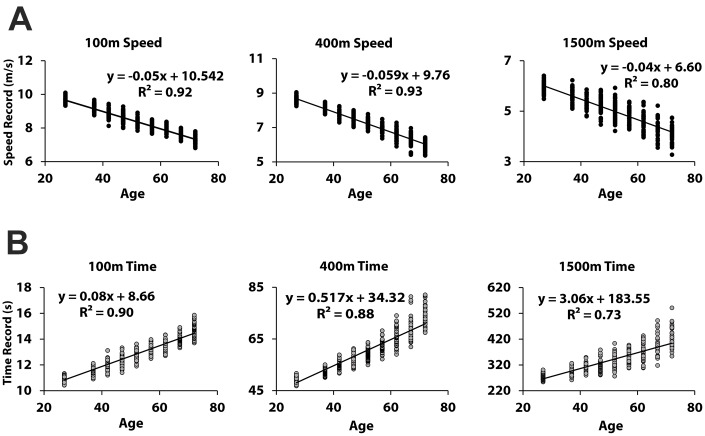
**Linear decrease in performance with increase in age.** (**A**) and (**B**) show the age-dependent linear decreases in speed and the linear increases in record times, respectively. The R^2^ values for the regression of the both variables on age are all high, but the regression of speed on age shows higher R^2^ values for all three events.

The performance of each age group in each of the three track events is individually shown in Figure 4. Analysis of variance (ANOVA) and post-hoc comparisons show statistically significant differences between all pairs of neighboring age groups in any event, except two pairs in the 1500m run: the pair of 40~44 and 45~49; and the pair of 60~64 and 65~69. The linearity between performance and age is additionally demonstrated in Figure 5. The R^2^ values for the regression of the average speeds on age are all close to unity. The R^2^ values for the regression of the record times on age are also fairly high but lower than those observed for average speed.

## DISCUSSION

A high value of a factor loading indicates a large contribution of specific discipline results, which refers to different motor abilities, to each PC. For example, high factor loadings of the 100m run imply that sprinting ability (strength) plays a dominant role in the formation of the PC, whereas high factor loadings of the 1500m run indicate the dominance of endurance. Figures 1, 2, and 3A clearly demonstrate that the relative contribution of sprinting ability and endurance to each PC, or the structural aspect of motor ability substantially changes due to aging. Importantly, most of the structural change develops around the age of 50. Regarding the temporal progression of aging, Lazarus and Harridge suggested that the age of 70 is the “breakpoint” beyond which the performance failure in masters athletics is likely to occur [22]. They argued that a decline in the synchrony and integration of the systems in charge of whole body performance emerges around this age. Wright and Perricelli also showed significant decreases in motor performance of elite senior athletes only after the age of 70s [20]. Considering these previous studies, our results suggest that the sudden change of the structure of motor ability can substantially precede the failure in the overall motor performance.

In particular, the dominant role of sprinting ability in the formation of the first PC diminishes after the age of 50. This finding is consistent with previously reported physiological data. Lexell et al. found that the number of motor units in the muscles of healthy adults remains almost constant until 50 years of age, but decreases almost linearly after 50 [23]. This critical physiological change can be partly responsible for the observed abrupt structural change of the motor ability. Another study shows that aging diminishes the performance of both marathon runners and weight-lifters, but the decline is faster for the weight-lifters [24]. The faster deterioration in performance in the power demanding sports is also consistent with the decreased role of sprinting ability in the first PC.

The step-like structural change in the motor ability contrasts sharply with the smooth decrease in motor performance itself. Figure 3B and Figure 5 clearly demonstrate that aging induces the gradual decrease in performance in each track event. The obvious contrast between the smooth decrease in the performance records and the sudden change in motor ability structure indicates a weak correlation between the performance and the motor ability structure. Although the apparent performance of athletes changes gradually, the underlying structure of motor ability may change abruptly. This finding further emphasizes the necessity of investigations of both the structural changes and overall performance deterioration; one cannot serve as a predictor of the other.

Statistical analysis shows significant differences between all pairs of neighboring age groups in the three track events, except two pairs in the 1500m run (Figure 4). On the other hand, Wright and Perricelli reported that the age at which performance first became significantly different from those of the previous age group was 70 years with regard to sprinting and 75 years with regard to endurance [20]. The result of our analysis differs from these findings. Both sprinting and endurance abilities of each age group, which are reflected in the records in 100m, 400m and 1500m run, are generally inferior to the performance of the previous age group with statistically significant difference.

The decrease in performance records is not only statistically significant but also almost linear with respect to age (Figure 5). The regression of both the record times and the average speeds on age shows high goodness of fit, but the R^2^ values for the regression of the average speeds on age are higher than those for the record times in all three track events. I.e., the linearity between age and speed is stronger than the linearity between age and record time. Mathematically, the average speed is inversely proportional to the time record in any race. For example, 10% increase in speed corresponds to 9.09% decrease in time, rather than 10% decrease. Twenty percent increase in speed corresponds to 16.7% decrease in time. This nonlinear relationship between time and speed should be responsible for the observed differences in the R^2^ values. The higher R^2^ values for the regression of the average speeds on age suggest that the average speed should be considered as the more reliable performance metric when we need to predict the performance of a specific age group e.g., by interpolation.

A limitation of this study is that all the confounding factors could not be controlled. However, we investigated the performance of only elite athletes (record holders) who maintained the similar, highest level of motor abilities by intense training. In addition, considering the large sample size and the diversity of the countries where the athletes have lived, the effect of the uncontrolled confounding factors is not expected to be significant. (The nationalities of the record holders are listed in the Supplementary Table 1).

The training strategy may enhance athletes’ performance further when the strategy is tuned according to the biological age of athletes. Multiple studies provided the evidence of efficacy of specialized training in enhancing specific motor ability of the elderly [25–27]. Our result shows that motor ability structure suddenly changes around the age of 50; the contribution of the sprinting ability to the formation of the first PC diminishes, and the sprinting ability begins to contribute to the second PC after 50. This finding suggests that a training strategy focusing more on sprinting ability around the age of 50 may decelerate the age-induced deterioration in decathlon performance. For example, significant increase in isometric force, dynamic force, and cross-sectional area of vastus lateralis muscle; and significant improvement in ground reaction force characteristics were obtained from 20 weeks strength and sprint training program for elite masters athletes [25]. Although the detailed strategy for effective training needs to be addressed carefully in the future work, the result of the current study provides important information regarding the age when the athletes should begin to pay more attention to power and sprinting training.

Not only the results but also the methods of this study are potentially useful. This is not the first study that adopted PCA in the field of sports science; various studies used PCA to extract major factors in the achievement of high performance in sports events [28, 29]. However, to the best of our knowledge, this is the first study that analyzed the age-dependent changes in the relative dominance of each motor ability by performing PCA across various age groups. In this initial study, we applied the method to decathlon in which the performance in each component event readily reflects each motor ability. However, various sports including tennis and water polo require multiple but almost independent motor abilities like strength, endurance, flexibility, etc. With proper database, we may perform PCA across various age groups for these sports events as we did in this study, which may contribute to understanding when and how the training strategy should be modified for the athletes to minimize the age-induced deterioration in performance.

## METHODS

### Subjects

We used publicly available data acquired from the results of the Summer Olympics held in 2000, 2004, 2008, 2012, and 2016; and the all-time male masters decathletes world records list from 1975 to 2012 without merging any of the data sets in such a way that individuals might be identified. In addition, this study did not enhance the public data set with identifiable, or potentially identifiable data, which concludes that no IRB approval is needed. We additionally obtained written confirmation that the data sets can be used for this study from the International Olympic Committee (IOC) Olympic Studies Centre, and the World Master Athletics, to which the ownership of the data sets belongs. All data used in this study are available from the following websites: www.iaaf.org, and www.mastersathletics.net. The data of any athlete with zero points in any of the ten events were excluded. We compiled the total of 467 rows of running records in the three track events (100m, 400m and 1500m runs), and then excluded 21 extreme outliers whose records were outside the three standard deviation intervals using a stem and leaf plot [30].

### Data analysis

Before performing PCA, each record (in time) was converted to average speed. For example, the record of 10.98 seconds in the 100m run was converted to 100 meters divided by 10.98 seconds, or 9.107 m/s. We chose to extract the first and the second PCs, consulting the results of previous decathlon studies [31, 32]. To identify PCs, we used the varimax rotation method for each age group separately, and the rotations converged in 3 iterations. We used one-way repeated-measures analysis of variance (ANOVA) to verify the differences in performance in each of the running events between different age groups with a significance level of 0.05. The Tukey honest significant difference (HSD) test was performed as a post hoc test with the same significance level. All statistical analyses were performed using SPSS for Windows Ver. 23 (SPSS, Inc., Chicago, IL, USA).

## Supplementary Material

Supplementary Table 1
