# Supplementary material for: Aging induces a step-like change in the motor ability structure of athletes
Source: Aging (Albany NY). 2019 Jul 30;11(14):5276–86. doi: 10.18632/aging.102126 (PMC6682536; doi:10.18632/aging.102126)
Supplement: Supplementary Table 1 [file aging-11-102126-s001.docx]

**Supplementary Table 1. Information of the athletes.**

|  | Multiple | Age Group | Nation | Dob | Venue | Date  Of Participation |
| --- | --- | --- | --- | --- | --- | --- |
| 1 |  | 20-34s | CZE | N/A | 2000 Sydney | 2000 |
| 2 |  | 20-34s | USA | N/A | 2000 Sydney | 2000 |
| 3 |  | 20-34s | USA | N/A | 2000 Sydney | 2000 |
| 4 |  | 20-34s | CZE | N/A | 2000 Sydney | 2000 |
| 5 |  | 20-34s | GER | N/A | 2000 Sydney | 2000 |
| 6 |  | 20-34s | HUN | N/A | 2000 Sydney | 2000 |
| 7 |  | 20-34s | GER | N/A | 2000 Sydney | 2000 |
| 8 |  | 20-34s | SWE | N/A | 2000 Sydney | 2000 |
| 9 |  | 20-34s | HUN | N/A | 2000 Sydney | 2000 |
| 10 |  | 20-34s | POR | N/A | 2000 Sydney | 2000 |
| 11 |  | 20-34s | RUS | N/A | 2000 Sydney | 2000 |
| 12 |  | 20-34s | CZE | N/A | 2000 Sydney | 2000 |
| 13 |  | 20-34s | CUB | N/A | 2000 Sydney | 2000 |
| 14 |  | 20-34s | UKR | N/A | 2000 Sydney | 2000 |
| 15 |  | 20-34s | EST | N/A | 2000 Sydney | 2000 |
| 16 |  | 20-34s | AUT | N/A | 2000 Sydney | 2000 |
| 17 |  | 20-34s | FRA | N/A | 2000 Sydney | 2000 |
| 18 |  | 20-34s | FRA | N/A | 2000 Sydney | 2000 |
| 19 |  | 20-34s | USA | N/A | 2000 Sydney | 2000 |
| 20 |  | 20-34s | UKR | N/A | 2000 Sydney | 2000 |
| 21 |  | 20-34s | UKR | N/A | 2000 Sydney | 2000 |
| 22 |  | 20-34s | FIN | N/A | 2000 Sydney | 2000 |
| 23 |  | 20-34s | CZE | N/A | 2004 Athens | 2004 |
| 24 |  | 20-34s | USA | N/A | 2004 Athens | 2004 |
| 25 |  | 20-34s | KAZ | N/A | 2004 Athens | 2004 |
| 26 |  | 20-34s | GBR | N/A | 2004 Athens | 2004 |
| 27 |  | 20-34s | NED | N/A | 2004 Athens | 2004 |
| 28 |  | 20-34s | HUN | N/A | 2004 Athens | 2004 |
| 29 |  | 20-34s | FRA | N/A | 2004 Athens | 2004 |
| 30 |  | 20-34s | EST | N/A | 2004 Athens | 2004 |
| 31 |  | 20-34s | JAM | N/A | 2004 Athens | 2004 |
| 32 |  | 20-34s | AUT | N/A | 2004 Athens | 2004 |
| 33 |  | 20-34s | RUS | N/A | 2004 Athens | 2004 |
| 34 |  | 20-34s | GER | N/A | 2004 Athens | 2004 |
| 35 |  | 20-34s | FRA | N/A | 2004 Athens | 2004 |
| 36 |  | 20-34s | JAM | N/A | 2004 Athens | 2004 |
| 37 |  | 20-34s | RUS | N/A | 2004 Athens | 2004 |
| 38 |  | 20-34s | FIN | N/A | 2004 Athens | 2004 |
| 39 |  | 20-34s | UZB | N/A | 2004 Athens | 2004 |
| 40 |  | 20-34s | CHN | N/A | 2004 Athens | 2004 |
| 41 |  | 20-34s | GER | N/A | 2004 Athens | 2004 |
| 42 |  | 20-34s | BLR | N/A | 2004 Athens | 2004 |
| 43 |  | 20-34s | USA | N/A | 2004 Athens | 2004 |
| 44 |  | 20-34s | SPA | N/A | 2004 Athens | 2004 |
| 45 |  | 20-34s | EST | N/A | 2004 Athens | 2004 |
| 46 |  | 20-34s | ARG | N/A | 2004 Athens | 2004 |
| 47 |  | 20-34s | LAT | N/A | 2004 Athens | 2004 |
| 48 |  | 20-34s | NOR | N/A | 2004 Athens | 2004 |
| 49 |  | 20-34s | BLR | N/A | 2008 Beijing | 2008 |
| 50 |  | 20-34s | CUB | N/A | 2008 Beijing | 2008 |
| 51 |  | 20-34s | FRA | N/A | 2008 Beijing | 2008 |
| 52 |  | 20-34s | CZE | N/A | 2008 Beijing | 2008 |
| 53 |  | 20-34s | UKR | N/A | 2008 Beijing | 2008 |
| 54 |  | 20-34s | GER | N/A | 2008 Beijing | 2008 |
| 55 |  | 20-34s | JAM | N/A | 2008 Beijing | 2008 |
| 56 |  | 20-34s | GER | N/A | 2008 Beijing | 2008 |
| 57 |  | 20-34s | EST | N/A | 2008 Beijing | 2008 |
| 58 |  | 20-34s | RUS | N/A | 2008 Beijing | 2008 |
| 59 |  | 20-34s | EST | N/A | 2008 Beijing | 2008 |
| 60 |  | 20-34s | NED | N/A | 2008 Beijing | 2008 |
| 61 |  | 20-34s | CUB | N/A | 2008 Beijing | 2008 |
| 62 |  | 20-34s | BLR | N/A | 2008 Beijing | 2008 |
| 63 |  | 20-34s | BLR | N/A | 2008 Beijing | 2008 |
| 64 |  | 20-34s | CHN | N/A | 2008 Beijing | 2008 |
| 65 |  | 20-34s | CAN | N/A | 2008 Beijing | 2008 |
| 66 |  | 20-34s | GBR | N/A | 2008 Beijing | 2008 |
| 67 |  | 20-34s | SLO | N/A | 2008 Beijing | 2008 |
| 68 |  | 20-34s | USA | N/A | 2012 London | 2012 |
| 69 |  | 20-34s | CUB | N/A | 2012 London | 2012 |
| 70 |  | 20-34s | BEL | N/A | 2012 London | 2012 |
| 71 |  | 20-34s | CAN | N/A | 2012 London | 2012 |
| 72 |  | 20-34s | GER | N/A | 2012 London | 2012 |
| 73 |  | 20-34s | UKR | N/A | 2012 London | 2012 |
| 74 |  | 20-34s | RUS | N/A | 2012 London | 2012 |
| 75 |  | 20-34s | RSA | N/A | 2012 London | 2012 |
| 76 |  | 20-34s | GER | N/A | 2012 London | 2012 |
| 77 |  | 20-34s | NED | N/A | 2012 London | 2012 |
| 78 |  | 20-34s | NZL | N/A | 2012 London | 2012 |
| 79 |  | 20-34s | CHI | N/A | 2012 London | 2012 |
| 80 |  | 20-34s | CUB | N/A | 2012 London | 2012 |
| 81 |  | 20-34s | FRA | N/A | 2012 London | 2012 |
| 82 |  | 20-34s | RUS | N/A | 2012 London | 2012 |
| 83 |  | 20-34s | BLR | N/A | 2012 London | 2012 |
| 84 |  | 20-34s | BRA | N/A | 2012 London | 2012 |
| 85 |  | 20-34s | JPN | N/A | 2012 London | 2012 |
| 86 |  | 20-34s | NED | N/A | 2012 London | 2012 |
| 87 |  | 20-34s | LAT | N/A | 2012 London | 2012 |
| 88 |  | 20-34s | HUN | N/A | 2012 London | 2012 |
| 89 |  | 20-34s | LTU | N/A | 2012 London | 2012 |
| 90 |  | 20-34s | FRA | N/A | 2016 Rio De Janeiro | 2016 |
| 91 |  | 20-34s | ALG | N/A | 2016 Rio De Janeiro | 2016 |
| 92 |  | 20-34s | CUB | N/A | 2016 Rio De Janeiro | 2016 |
| 93 |  | 20-34s | USA | N/A | 2016 Rio De Janeiro | 2016 |
| 94 |  | 20-34s | BEL | N/A | 2016 Rio De Janeiro | 2016 |
| 95 |  | 20-34s | GRN | N/A | 2016 Rio De Janeiro | 2016 |
| 96 |  | 20-34s | BRA | N/A | 2016 Rio De Janeiro | 2016 |
| 97 |  | 20-34s | USA | N/A | 2016 Rio De Janeiro | 2016 |
| 98 |  | 20-34s | CZE | N/A | 2016 Rio De Janeiro | 2016 |
| 99 |  | 20-34s | FRA | N/A | 2016 Rio De Janeiro | 2016 |
| 100 |  | 20-34s | AUS | N/A | 2016 Rio De Janeiro | 2016 |
| 101 |  | 20-34s | GER | N/A | 2016 Rio De Janeiro | 2016 |
| 102 |  | 20-34s | GRN | N/A | 2016 Rio De Janeiro | 2016 |
| 103 |  | 20-34s | SPA | N/A | 2016 Rio De Janeiro | 2016 |
| 104 |  | 20-34s | CUB | N/A | 2016 Rio De Janeiro | 2016 |
| 105 |  | 20-34s | AUT | N/A | 2016 Rio De Janeiro | 2016 |
| 106 |  | 20-34s | JPN | N/A | 2016 Rio De Janeiro | 2016 |
| 107 |  | 20-34s | POL | N/A | 2016 Rio De Janeiro | 2016 |
| 108 |  | 20-34s | JPN | N/A | 2016 Rio De Janeiro | 2016 |
| 109 |  | 20-34s | EST | N/A | 2016 Rio De Janeiro | 2016 |
| 110 |  | 35-39 | RUS | 01.04.1969 | Götzis | 30.05.2004 |
| 111 |  | 35-39 | CZE | 26.11.1974 | Albi | 29.07.2011 |
| 112 |  | 35-39 | CZE | 11.05.1972 | Kladno | 20.06.2007 |
| 113 |  | 35-39 | RUS | 11.08.1965 | Tula | 13.07.2001 |
| 114 |  | 35-39 | EST | 16.12.1967 | Kärdla | 10.08.2003 |
| 115 |  | 35-39 | HUN | 29.04.1977 | Budapest | 16.09.2012 |
| 116 |  | 35-39 | FRA | 11.04.1969 | Talence | 18.09.2005 |
| 117 | Multiple | 35-39 | NZL | 27.05.1963 | Kuala Lumpur | 18.08.1998 |
| 118 |  | 35-39 | GER | 24.05.1936 | Bonn | 12.05.1972 |
| 119 |  | 35-39 | SWE | 18.05.1944 | Stockholm | 08.08.1982 |
| 120 | Multiple | 35-39 | NZL | 09.09.1934 | Los Angeles | 08.07.1970 |
| 121 | Multiple | 35-39 | AUT | 08.01.1936 | Schielleiten | 03.06.1972 |
| 122 |  | 35-39 | GER | 30.04.1943 | Ingelheim | 18.08.1979 |
| 123 |  | 35-39 | RUS | 25.07.1937 | Dnepropetrovsk | 17.09.1972 |
| 124 | Multiple | 35-39 | GER | 05.11.1940 | Santa Barbara | 31.03.1976 |
| 125 |  | 35-39 | VEN | 01.10.1963 | Bogota | 26.06.1999 |
| 126 | Multiple | 35-39 | AUT | 07.04.1956 | Ebensee | 25.09.1991 |
| 127 |  | 35-39 | SWE | 07.06.1971 | Enköping | 15.07.2007 |
| 128 |  | 35-39 | NZL | 17.06.1962 | Auckland | 28.03.1999 |
| 129 |  | 35-39 | IRL | 23.12.1955 | Wrexham | 26.05.1991 |
| 130 |  | 35-39 | HUN | 16.06.1954 | Budapest | 22.07.1990 |
| 131 |  | 35-39 | GER | 11.03.1968 | Vaterstetten | 04.09.2004 |
| 132 | Multiple | 35-39 | GBR | 18.11.1963 | Stoke-on-Trent | 30.07.2000 |
| 133 |  | 35-39 | ESP | 22.09.1943 | Barcelona | 29.07.1979 |
| 134 |  | 35-39 | FIN | 31.08.1946 | Turku | 27.09.1981 |
| 135 | Multiple | 35-39 | GER | 19.02.1944 | Göttingen | 13.07.1979 |
| 136 |  | 35-39 | MOR | 22.06.1971 | Addis Ababa | 30.04.2008 |
| 137 | Multiple | 35-39 | USA | 06.07.1946 | Lincoln | 20.06.1982 |
| 138 | Multiple | 35-39 | GBR | 15.04.1948 | Birmingham | 10.07.1983 |
| 139 |  | 35-39 | ITA | 12.01.1969 | Ostia-Roma | 12.09.2004 |
| 140 |  | 35-39 | ESP | 08.08.1976 | Arona | 27.05.2012 |
| 141 |  | 35-39 | USA | 11.12.1950 | Boulder | 11.07.1987 |
| 142 | Multiple | 35-39 | GER | 10.09.1966 | Vaterstetten | 04.09.2004 |
| 143 |  | 35-39 | FRA | 26.12.1969 | San Sebastian | 23.08.2005 |
| 144 | Multiple | 35-39 | CZE | 04.02.1944 | Praha | 08.06.1980 |
| 145 |  | 35-39 | GER | 27.07.1944 | Leverkusen | 16.06.1980 |
| 146 | Multiple | 35-39 | DEN | 28.04.1953 | Copenhagen | 20.08.1989 |
| 147 |  | 35-39 | GER | 06.12.1940 | Munich | 26.09.1976 |
| 148 | Multiple | 35-39 | GER | 08.09.1938 | Leverkusen | 16.06.1980 |
| 149 |  | 35-39 | CAN | 1946 | San Diego | 05.07.1985 |
| 150 |  | 35-39 | GER | 28.01.1942 | Essen | 10.06.1978 |
| 151 | Multiple | 35-39 | GER | 04.07.1967 | Leipzig | 11.05.2003 |
| 152 |  | 35-39 | NED | 1976 | Zittau | 16.08.2012 |
| 153 |  | 35-39 | SWE | 27.09.1970 | Huddersfield | 11.09.2010 |
| 154 | Multiple | 40-44 | NZL | 27.05.1963 | Osnabrück | 26.06.2004 |
| 155 |  | 40-44 | GBR | 25.04.1956 | Enfield | 02.08.1997 |
| 156 |  | 40-44 | USA | 27.07.1964 | Carthage | 21.08.2004 |
| 157 | Multiple | 40-44 | GER | 05.11.1940 | San Antonio | 07.06.1981 |
| 158 | Multiple | 40-44 | AUT | 08.01.1936 | Bludenz | 04.09.1976 |
| 159 | Multiple | 40-44 | GER | 08.09.1938 | Gelsenkirchen | 07.10.1978 |
| 160 | Multiple | 40-44 | ITA | 24.03.1957 | Garmisch | 07.06.1997 |
| 161 |  | 40-44 | USA | 17.03.1955 |  | 20.09.1997 |
| 162 | Multiple | 40-44 | USA | 06.07.1946 | Eugene | 27.07.1989 |
| 163 |  | 40-44 | ISL | 09.06.1934 | Reykjavik | 06.09.1975 |
| 164 | Multiple | 40-44 | AUT | 07.04.1956 | Traun | 26.09.1999 |
| 165 |  | 40-44 | USA | 06.05.1941 | San Antonio | 07.06.1981 |
| 166 | Multiple | 40-44 | NZL | 09.09.1934 |  | 06.06.1975 |
| 167 | Multiple | 40-44 | CZE | 04.02.1944 | Praha | 1985 |
| 168 | Multiple | 40-44 | GER | 10.09.1966 | Viernheim | 02.07.2011 |
| 169 |  | 40-44 | GER | 1959 | Gateshead | 29.07.1999 |
| 170 | Multiple | 40-44 | GER | 04.07.1967 | Niesky | 21.07.2007 |
| 171 | Multiple | 40-44 | BEL | 15.07.1949 | Turku | 19.07.1991 |
| 172 |  | 40-44 | ESP | 20.01.1967 | Alhama de Murcia | 31.05.2009 |
| 173 | Multiple | 40-44 | GBR | 15.04.1948 | Midland | 01.05.1988 |
| 174 | Multiple | 40-44 | USA | 24.08.1933 | Santa Maria | 05.07.1975 |
| 175 |  | 40-44 | USA | 04.08.1958 | Trenton | 22.07.2002 |
| 176 | Multiple | 40-44 | GER | 14.07.1954 | Vienna | 10.06.1995 |
| 177 | Multiple | 40-44 | GBR | 18.11.1963 | Riccione | 04.09.2007 |
| 178 |  | 40-44 | FIN | 10.04.1951 | Turku | 19.07.1991 |
| 179 |  | 40-44 | GER | 24.07.1969 | Lahti | 28.07.2009 |
| 180 | Multiple | 40-44 | GER | 28.03.1955 | Buffalo | 13.07.1995 |
| 181 | Multiple | 40-44 | DEN | 01.04.1955 | Herlufsholm | 28.06.1996 |
| 182 | Multiple | 40-44 | USA | 01.12.1943 | Uniondale | 18.07.1986 |
| 183 |  | 40-44 | GER | 1964 | Arhus | 23.07.2004 |
| 184 |  | 40-44 | NED | 05.02.1959 | Vught | 30.09.2001 |
| 185 |  | 40-44 | GRE | 21.08.1967 | Ljubljana | 24.07.2008 |
| 186 | Multiple | 40-44 | GER | 30.04.1967 | Nyiregyhaza | 15.07.2010 |
| 187 | Multiple | 40-44 | USA | 1933 |  | 02.08.1974 |
| 188 |  | 45-49 | RUS | 19.12.1951 | Durban | 17.07.1997 |
| 189 |  | 45-49 | ISL | 09.06.1934 | Reykjavik | 09.09.1979 |
| 190 | Multiple | 45-49 | GER | 10.09.1966 | Zittau | 16.08.2012 |
| 191 | Multiple | 45-49 | GER | 30.04.1967 | Zittau | 16.08.2012 |
| 192 | Multiple | 45-49 | NZL | 09.09.1934 | Auckland | 07.02.1981 |
| 193 | Multiple | 45-49 | AUT | 07.04.1956 | Linz | 29.09.2001 |
| 194 | Multiple | 45-49 | ITA | 24.03.1957 | Lana | 28.04.2002 |
| 195 | Multiple | 45-49 | USA | 06.07.1946 | Turku | 18.07.1991 |
| 196 | Multiple | 45-49 | USA | 07.01.1932 | Birmingham | 12.08.1978 |
| 197 |  | 45-49 | USA | 07.06.1964 | Joplin | 20.06.2010 |
| 198 |  | 45-49 | FRA | 15.01.1963 | Compiegne | 07.06.2008 |
| 199 | Multiple | 45-49 | GER | 14.07.1954 | Gateshead | 29.07.1999 |
| 200 |  | 45-49 | AUT | 30.06.1966 | Zittau | 16.08.2012 |
| 201 |  | 45-49 | PUR | 22.09.1964 | Sacramento | 07.07.2011 |
| 202 |  | 45-49 | USA | 10.10.1937 | Los Angeles | 07.07.1984 |
| 203 | Multiple | 45-49 | GBR | 18.11.1963 | Lahti | 28.07.2009 |
| 204 | Multiple | 45-49 | USA | 02.07.1937 | San Diego | 05.07.1985 |
| 205 | Multiple | 45-49 | GBR | 20.06.1947 | Miyazaki | 07.10.1993 |
| 206 |  | 45-49 | USA | 24.10.1957 | Carolina | 02.07.2003 |
| 207 | Multiple | 45-49 | USA | 24.08.1933 | San Diego | 14.07.1979 |
| 208 | Multiple | 45-49 | NOR | 27.08.1949 | Buffalo | 13.07.1995 |
| 209 | Multiple | 45-49 | GER | 07.02.1940 | Eugene | 27.07.1989 |
| 210 | Multiple | 45-49 | USA | 29.10.1937 | Indianapolis | 07.07.1984 |
| 211 |  | 45-49 | GBR | 08.04.1952 | Durban | 17.07.1997 |
| 212 | Multiple | 45-49 | DEN | 28.04.1953 | Gateshead | 29.07.1999 |
| 213 | Multiple | 45-49 | USA | 13.08.1957 | Carolina | 03.07.2003 |
| 214 |  | 45-49 | GER | 22.04.1957 | Carolina | 03.07.2003 |
| 215 |  | 45-49 | FRA | 04.10.1959 | San Sebastian | 23.08.2005 |
| 216 | Multiple | 45-49 | GER | 30.07.1960 | San Sebastian | 23.08.2005 |
| 217 | Multiple | 45-49 | GER | 22.02.1962 | Lahti | 29.07.2009 |
| 218 | Multiple | 45-49 | GER | 25.12.1938 | Göttingen | 11.06.1984 |
| 219 |  | 45-49 | GER | 1943 | Turku | 18.07.1991 |
| 220 | Multiple | 45-49 | GER | 1950 | Durban | 17.07.1997 |
| 221 |  | 45-49 | GER | 22.03.1960 | Poznan | 20.07.2006 |
| 222 | Multiple | 45-49 | GER | 22.09.1959 | Riccione | 04.09.2007 |
| 223 |  | 45-49 | GER | 1967 | Zittau | 16.08.2012 |
| 224 | Multiple | 45-49 | NOR | 10.01.1937 | Kil | 25.09.1982 |
| 225 |  | 45-49 | SUI | 15.03.1946 | Turku | 18.07.1991 |
| 226 |  | 45-49 | AUT | 1967 | Wien | 30.09.2012 |
| 227 | Multiple | 45-49 | USA | 05.12.1949 | Buffalo | 13.07.1995 |
| 228 |  | 45-49 | POL | 14.07.1963 | Sacramento | 07.07.2011 |
| 229 |  | 45-49 | JPN | 22.07.1964 | Ishikawa | 30.10.2011 |
| 230 |  | 45-49 | NED | 26.07.1954 | Berlin | 16.07.2000 |
| 231 |  | 45-49 | AUT | 05.01.1961 | Ljubljana | 24.07.2008 |
| 232 |  | 45-49 | LTU | 26.04.1962 | Lignano | 14.09.2011 |
| 233 | Multiple | 45-49 | GBR | 23.07.1960 | Oxford | 12.08.2006 |
| 234 | Multiple | 45-49 | GBR | 27.07.1957 | Carolina | 02.07.2003 |
| 235 |  | 45-49 | GBR | 14.12.1935 | Surrey | 19.06.1982 |
| 236 |  | 45-49 | GER | 1966 | Zittau | 16.08.2012 |
| 237 | Multiple | 45-49 | GBR | 15.04.1948 | Sheffield | 11.09.1993 |
| 238 | Multiple | 45-49 | LTU | 22.01.1959 | San Sebastian | 23.08.2005 |
| 239 | Multiple | 45-49 | GER | 05.01.1940 | Zeven | 20.09.1986 |
| 240 |  | 45-49 | USA | 18.03.1964 | Lahti | 29.07.2009 |
| 241 | Multiple | 45-49 | GBR | 27.05.1958 | Sheffield | 13.09.2003 |
| 242 | Multiple | 45-49 | GBR | 09.10.1948 | Buffalo | 13.07.1995 |
| 243 |  | 45-49 | USA | 1943 | Eugene | 28.07.1989 |
| 244 | Multiple | 45-49 | NED | 05.01.1952 | Brisbane | 06.07.2001 |
| 245 | Multiple | 50-54 | GER | 19.02.1944 | Göttingen | 29.06.1996 |
| 246 | Multiple | 50-54 | ITA | 24.03.1957 | Milano | 02.07.2007 |
| 247 | Multiple | 50-54 | AUT | 07.04.1956 | Poznan | 20.07.2006 |
| 248 | Multiple | 50-54 | BEL | 15.07.1949 | Gateshead | 29.07.1999 |
| 249 | Multiple | 50-54 | GER | 07.02.1940 | Turku | 20.07.1991 |
| 250 |  | 50-54 | GER | 07.01.1953 | Zeven | 13.09.2003 |
| 251 | Multiple | 50-54 | NED | 05.01.1952 | Carolina | 03.07.2003 |
| 252 |  | 50-54 | GER | 16.02.1939 | Berlin | 24.08.1991 |
| 253 | Multiple | 50-54 | GER | 28.03.1955 | San Sebastian | 23.08.2005 |
| 254 | Multiple | 50-54 | GER | 30.07.1960 | Lignano | 14.09.2011 |
| 255 | Multiple | 50-54 | USA | 01.05.1921 | Honolulu | 11.03.1972 |
| 256 |  | 50-54 | USA | 01.04.1950 | Neosha | 29.07.2000 |
| 257 | Multiple | 50-54 | USA | 06.01.1954 | Seattle | 16.07.2006 |
| 258 |  | 50-54 | USA | 06.08.1956 | Riccione | 04.09.2007 |
| 259 | Multiple | 50-54 | USA | 1933 | Merced | 03.07.1983 |
| 260 |  | 50-54 | FIN | 05.11.1940 | Turku | 19.07.1991 |
| 261 | Multiple | 50-54 | GER | 22.02.1962 | Zittau | 16.08.2012 |
| 262 | Multiple | 50-54 | GER | 08.09.1938 | Turku | 20.07.1991 |
| 263 | Multiple | 50-54 | LTU | 22.01.1959 | Lignano | 14.09.2011 |
| 264 | Multiple | 50-54 | GER | 22.09.1959 | Dillingen | 15.05.2010 |
| 265 | Multiple | 50-54 | GER | 14.07.1954 | Arhus | 23.07.2004 |
| 266 | Multiple | 50-54 | GER | 1950 | Brisbane | 04.07.2001 |
| 267 | Multiple | 50-54 | LTU | 22.01.1959 | Lahti | 28.07.2009 |
| 268 | Multiple | 50-54 | GBR | 20.06.1947 | Gateshead | 29.07.1999 |
| 269 | Multiple | 50-54 | GBR | 27.07.1957 | Riccione | 04.09.2008 |
| 270 | Multiple | 50-54 | USA | 02.07.1937 | Eugene | 28.07.1989 |
| 271 |  | 50-54 | USA | 1939 | Eugene | 28.07.1989 |
| 272 | Multiple | 50-54 | USA | 13.08.1957 | Joplin | 20.06.2010 |
| 273 |  | 50-54 | SVK | 24.07.1957 | Ljubljana | 24.07.2008 |
| 274 |  | 50-54 | GER | 1939 | Eugene | 28.07.1989 |
| 275 |  | 50-54 | USA | 19.08.1944 | Buffalo | 13.07.1995 |
| 276 |  | 50-54 | CAN | 24.05.1953 | San Sebastian | 23.08.2005 |
| 277 | Multiple | 50-54 | USA | 06.07.1946 | San Diego | 06.07.1996 |
| 278 | Multiple | 50-54 | GER | 24.09.1947 | Gateshead | 29.07.1999 |
| 279 |  | 50-54 | GER | 08.08.1958 | Ahlen | 13.09.2008 |
| 280 | Multiple | 50-54 | GER | 13.11.1938 | Warstein | 28.09.1991 |
| 281 | Multiple | 50-54 | GBR | 09.10.1948 | Brisbane | 06.07.2001 |
| 282 |  | 50-54 | GER | 1954 | Arhus | 22.07.2004 |
| 283 | Multiple | 50-54 | USA | 29.10.1937 | Thomasville | 18.06.1989 |
| 284 | Multiple | 50-54 | GBR | 15.04.1948 | Sheffield | 09.09.2000 |
| 285 | Multiple | 50-54 | GBR | 27.05.1958 | Ljubljana | 24.07.2008 |
| 286 | Multiple | 50-54 | NOR | 10.01.1937 | Oslo | 06.09.1987 |
| 287 |  | 50-54 | POL | 07.01.1951 | Brisbane | 06.07.2001 |
| 288 | Multiple | 50-54 | GER | 08.11.1943 | Buffalo | 13.07.1995 |
| 289 | Multiple | 50-54 | NOR | 27.08.1949 | Brisbane | 06.07.2001 |
| 290 |  | 50-54 | SWE | 24.02.1943 | Frederiksberg | 28.06.1996 |
| 291 |  | 50-54 | CAN | 17.11.1951 | San Sebastian | 23.08.2005 |
| 292 |  | 50-54 | POL | 22.06.1956 | Ljubljana | 24.07.2008 |
| 293 |  | 50-54 | GER | 1945 | Buffalo | 13.07.1995 |
| 294 | Multiple | 50-54 | USA | 05.12.1949 | Charlotte | 14.06.2003 |
| 295 | Multiple | 50-54 | GER | 05.01.1940 | Turku | 20.07.1991 |
| 296 | Multiple | 50-54 | GBR | 23.07.1960 | Sacramento | 07.07.2011 |
| 297 | Multiple | 55-59 | USA | 01.05.1921 | Gresham | 11.07.1976 |
| 298 | Multiple | 55-59 | USA | 06.01.1954 | Lahti | 28.07.2009 |
| 299 | Multiple | 55-59 | AUT | 07.04.1956 | Lignano | 14.09.2011 |
| 300 | Multiple | 55-59 | USA | 29.10.1937 | Buffalo | 13.07.1995 |
| 301 | Multiple | 55-59 | USA | 07.01.1933 | Los Angeles | 09.07.1988 |
| 302 | Multiple | 55-59 | GER | 13.11.1938 | Dortmund | 14.05.1994 |
| 303 |  | 55-59 | FIN | 24.03.1957 | Zittau | 16.08.2012 |
| 304 | Multiple | 55-59 | GBR | 20.06.1947 | Sheffield | 11.09.2004 |
| 305 | Multiple | 55-59 | GER | 08.09.1938 | Miyazaki | 07.10.1993 |
| 306 | Multiple | 55-59 | FIN | 15.10.1933 | Turku | 19.07.1991 |
| 307 |  | 55-59 | SLO | 26.04.1936 | Turku | 19.07.1991 |
| 308 |  | 55-59 | ISL | 16.07.1948 | Arhus | 03.07.2004 |
| 309 | Multiple | 55-59 | GER | 24.09.1947 | Carolina | 02.07.2003 |
| 310 | Multiple | 55-59 | CAN | 08.06.1949 | Shoreline | 15.07.2006 |
| 311 | Multiple | 55-59 | GER | 1937 | Miyazaki | 07.10.1993 |
| 312 |  | 55-59 | USA | 05.02.1926 | San Diego | 28.08.1982 |
| 313 | Multiple | 55-59 | USA | 14.01.1939 | Neosho | 25.07.1998 |
| 314 | Multiple | 55-59 | GER | 14.07.1954 | Lahti | 28.07.2009 |
| 315 | Multiple | 55-59 | USA | 1946 | Carolina | 03.07.2003 |
| 316 | Multiple | 55-59 | NOR | 10.01.1937 |  | 15.07.1992 |
| 317 |  | 55-59 | SVK | 24.07.1957 | Zittau | 16.08.2012 |
| 318 | Multiple | 55-59 | GER | 08.11.1943 | Gateshead | 29.07.1999 |
| 319 | Multiple | 55-59 | USA | 02.07.1937 | Miyazaki | 07.10.1993 |
| 320 |  | 55-59 | GBR | 09.12.1937 | Miyazaki | 07.10.1993 |
| 321 |  | 55-59 | USA | 12.11.1952 | Joplin | 21.06.2008 |
| 322 |  | 55-59 | USA | 04.07.1934 | Turku | 19.07.1991 |
| 323 | Multiple | 55-59 | NED | 05.01.1952 | Riccione | 04.09.2007 |
| 324 |  | 55-59 | GER | 06.04.1940 | Flein | 11.10.1997 |
| 325 | Multiple | 55-59 | RUS | 15.03.1943 | Gateshead | 29.07.1999 |
| 326 |  | 55-59 | POL | 1955 | Zittau | 16.08.2012 |
| 327 | Multiple | 55-59 | GBR | 1957 | Zittau | 16.08.2012 |
| 328 |  | 55-59 | JPN | 1951 | Ishikawa | 2006 |
| 329 | Multiple | 55-59 | GBR | 15.04.1948 | Sheffield | 11.09.2004 |
| 330 | Multiple | 55-59 | SWE | 16.11.1935 | Miyazaki | 07.10.1993 |
| 331 | Multiple | 55-59 | JPN | 1938 | Miyazaki | 07.10.1993 |
| 332 |  | 55-59 | USA | 18.12.1952 | Joplin | 21.06.2008 |
| 333 |  | 55-59 | GER | 21.07.1940 | Gildehaus | 11.10.1995 |
| 334 |  | 55-59 | FIN | 17.04.1954 | Lahti | 28.07.2009 |
| 335 |  | 55-59 | USA | 08.02.1945 | Trenton | 22.06.2002 |
| 336 |  | 55-59 | EST | 17.08.1951 | Lahti | 28.07.2009 |
| 337 | Multiple | 60-64 | GER | 19.02.1944 | San Sebastian | 23.08.2005 |
| 338 | Multiple | 60-64 | USA | 07.01.1933 | Miyazaki | 07.10.1993 |
| 339 | Multiple | 60-64 | USA | 14.01.1939 | Grass Valley | 03.07.1999 |
| 340 |  | 60-64 | NED | 26.02.1952 | Zittau | 16.08.2012 |
| 341 | Multiple | 60-64 | USA | 01.05.1921 | San Diego | 29.08.1982 |
| 342 | Multiple | 60-64 | GER | 1937 | Obertshausen | 04.05.1997 |
| 343 |  | 60-64 | USA | 22.06.1915 | Glendale | 06.12.1975 |
| 344 |  | 60-64 | GER | 17.03.1949 | Sacramento | 07.07.2011 |
| 345 | Multiple | 60-64 | CAN | 06.03.1946 | Seattle | 15.07.2006 |
| 346 | Multiple | 60-64 | GER | 13.11.1938 | Gladbeck | 14.09.2002 |
| 347 |  | 60-64 | LAT | 01.02.1948 | Ljubljana | 24.07.2008 |
| 348 |  | 60-64 | GER | 12.02.1944 | Arhus | 22.07.2004 |
| 349 | Multiple | 60-64 | NOR | 10.01.1937 | Oslo | 12.09.1997 |
| 350 |  | 60-64 | GBR | 25.04.1938 | Glasgow | 02.05.1998 |
| 351 |  | 60-64 | USA | 07.08.1940 | Brisbane | 04.07.2001 |
| 352 | Multiple | 60-64 | GBR | 20.06.1947 | Lahti | 28.07.2009 |
| 353 |  | 60-64 | USA | 28.04.1911 |  | 17.05.1974 |
| 354 | Multiple | 60-64 | GER | 01.05.1939 | Hamburg | 02.10.1999 |
| 355 | Multiple | 60-64 | JPN | 1942 | Ishikawa | 01.10.2004 |
| 356 | Multiple | 60-64 | GER | 25.12.1938 | Göttingen | 03.07.1999 |
| 357 |  | 60-64 | AUT | 27.01.1948 | Ljubljana | 24.07.2008 |
| 358 | Multiple | 60-64 | GER | 08.09.1938 | Leverkusen | 02.10.1999 |
| 359 |  | 60-64 | FIN | 04.07.1949 | Helsinki | 16.09.2012 |
| 360 |  | 60-64 | AUS | 26.10.1926 | Eugene | 28.07.1989 |
| 361 | Multiple | 60-64 | CAN | 08.06.1949 | Joplin | 20.06.2010 |
| 362 |  | 60-64 | NOR | 31.07.1936 | Moss | 23.08.1997 |
| 363 | Multiple | 60-64 | JPN | 1942 | Osaka | 24.10.2004 |
| 364 | Multiple | 60-64 | SWE | 16.11.1935 | Frederiksberg | 28.06.1996 |
| 365 |  | 60-64 | USA | 14.01.1943 | Carolina | 03.07.2003 |
| 366 | Multiple | 60-64 | USA | 01.12.1943 | Hoover | 09.06.2007 |
| 367 |  | 60-64 | LTU | 22.04.1947 | Malmö | 30.09.2008 |
| 368 | Multiple | 60-64 | RUS | 15.03.1943 | Arhus | 22.07.2004 |
| 369 |  | 60-64 | SWE | 05.03.1926 | Eugene | 28.07.1989 |
| 370 |  | 60-64 | GBR | 10.07.1941 | Sheffield | 20.07.2002 |
| 371 |  | 60-64 | USA | 1927 | Eugene | 28.07.1989 |
| 372 |  | 60-64 | SUI | 15.03.1946 | Poznan | 21.07.2006 |
| 373 | Multiple | 60-64 | USA | 05.12.1949 | Lynchburg | 25.09.2010 |
| 374 |  | 60-64 | USA | 30.08.1942 | Carthage | 21.08.2004 |
| 375 |  | 60-64 | FIN | 19.09.1927 | Turku | 19.07.1991 |
| 376 | Multiple | 60-64 | USA | 02.10.1926 | Thomasville | 18.06.1989 |
| 377 | Multiple | 60-64 | GER | 15.01.1946 | Erfurt | 19.08.2006 |
| 378 |  | 60-64 | GER | 1931 | Turku | 19.07.1991 |
| 379 | Multiple | 60-64 | EST | 19.07.1947 | Lahti | 28.07.2009 |
| 380 |  | 60-64 | FIN | 23.02.1926 | Eugene | 28.07.1989 |
| 381 | Multiple | 65-69 | GER | 18.02.1944 | Lahti | 28.07.2009 |
| 382 | Multiple | 65-69 | USA | 01.05.1921 | Boulder | 12.07.1987 |
| 383 | Multiple | 65-69 | USA | 14.01.1939 | San Sebastian | 23.08.2005 |
| 384 | Multiple | 65-69 | GER | 1946 | Zittau | 16.08.2012 |
| 385 | Multiple | 65-69 | NOR | 10.01.1937 | Carolina | 03.07.2003 |
| 386 | Multiple | 65-69 | GER | 01.05.1939 | Arhus | 24.07.2004 |
| 387 |  | 65-69 | FIN | 19.03.1924 | Eugene | 27.07.1989 |
| 388 |  | 65-69 | ESP | 04.12.1943 | Alhama de Murcia | 31.05.2009 |
| 389 | Multiple | 65-69 | GER | 06.11.1938 | San Sebastian | 23.08.2005 |
| 390 | Multiple | 65-69 | SWE | 16.11.1935 | Brisbane | 06.07.2001 |
| 391 |  | 65-69 | CAN | 20.08.1914 | Hillsdale | 20.06.1981 |
| 392 | Multiple | 65-69 | JPN | 1938 | Ishikawa | 01.10.2004 |
| 393 | Multiple | 65-69 | USA | 02.10.1926 | Thomasville | 20.09.1992 |
| 394 |  | 65-69 | AUS | 27.12.1923 | Eugene | 28.07.1989 |
| 395 | Multiple | 65-69 | GER | 13.11.1938 | Arhus | 24.07.2004 |
| 396 | Multiple | 65-69 | PUR | 18.06.1913 | San Juan | 31.07.1982 |
| 397 | Multiple | 65-69 | POL | 08.07.1941 | Poznan | 20.07.2006 |
| 398 | Multiple | 65-69 | AUS | 12.02.1939 | Brisbane | 19.02.2005 |
| 399 |  | 65-69 | USA | 18.07.1928 | Buffalo | 13.07.1995 |
| 400 |  | 65-69 | GER | 05.08.1945 | Arnsberg | 10.09.2010 |
| 401 | Multiple | 65-69 | EST | 1947 | Zittau | 16.08.2012 |
| 402 | Multiple | 65-69 | SWE | 28.10.1934 | Carolina | 03.07.2003 |
| 403 |  | 65-69 | CHI | 12.09.1929 | Buffalo | 13.07.1995 |
| 404 |  | 65-69 | USA | 09.04.1931 | Thomasville | 20.09.1997 |
| 405 | Multiple | 65-69 | NOR | 31.07.1936 | Carolina | 03.07.2003 |
| 406 | Multiple | 65-69 | CZE | 07.03.1933 | Gateshead | 29.07.1999 |
| 407 | Multiple | 65-69 | GER | 11.04.1925 | Flein | 30.09.1990 |
| 408 |  | 65-69 | RUS | 1944 | Lignano | 14.09.2011 |
| 409 | Multiple | 65-69 | FIN | 15.10.1933 | Gateshead | 29.07.1999 |
| 410 |  | 70-74 | NOR | 18.04.1921 | Turku | 19.07.1991 |
| 411 |  | 70-74 | USA | 22.04.1935 | Mayfield | 02.07.2005 |
| 412 |  | 70-74 | FIN | 07.08.1932 | Luumaki | 09.07.2005 |
| 413 | Multiple | 70-74 | NOR | 10.01.1937 | Hammerfest | 28.07.2007 |
| 414 | Multiple | 70-74 | USA | 14.01.1939 | Lahti | 28.07.2009 |
| 415 | Multiple | 70-74 | SWE | 28.10.1934 | Poznan | 20.07.2006 |
| 416 | Multiple | 70-74 | GER | 06.11.1938 | Lahti | 28.07.2009 |
| 417 |  | 70-74 | USA | 1937 | Hoover | 09.06.2007 |
| 418 | Multiple | 70-74 | USA | 01.05.1921 | Lincoln | 23.06.1991 |
| 419 |  | 70-74 | TUR | 01.06.1932 | Carolina | 03.07.2003 |
| 420 | Multiple | 70-74 | GER | 13.11.1938 | Ahlen | 12.09.2009 |
| 421 | Multiple | 70-74 | SWE | 16.11.1935 | Poznan | 20.07.2006 |
| 422 | Multiple | 70-74 | NOR | 31.07.1936 | Overhalla | 23.08.2008 |
| 423 | Multiple | 70-74 | CZE | 07.03.1933 | San Sebastian | 23.08.2005 |
| 424 | Multiple | 70-74 | GER | 01.05.1939 | Zittau | 16.08.2012 |
| 425 |  | 70-74 | USA | 1917 | Boulder | 12.07.1987 |
| 426 |  | 70-74 | USA | 21.06.1912 | San Diego | 28.08.1982 |
| 427 | Multiple | 70-74 | POL | 08.07.1941 | Lignano | 14.09.2011 |
| 428 |  | 70-74 | FIN | 04.06.1941 | Sacramento | 07.07.2011 |
| 429 |  | 70-74 | GER | 13.06.1938 | Ljubljana | 24.07.2008 |
| 430 |  | 70-74 | USA | 30.03.1933 | Hoover | 09.06.2007 |
| 431 | Multiple | 70-74 | PUR | 18.06.1913 | Indianapolis | 07.07.1984 |
| 432 |  | 70-74 | USA | 1912 | San Diego | 28.08.1982 |
| 433 | Multiple | 70-74 | AUS | 12.02.1939 | Brisbane | 28.02.2009 |
| 434 | Multiple | 70-74 | GER | 11.04.1925 | Flein | 13.10.1996 |
| 435 | Multiple | 70-74 | JPN | 1938 | Ishikawa | 03.10.2010 |
| 436 |  | 70-74 | DEN | 14.05.1941 | Sacramento | 07.07.2011 |
| 437 |  | 70-74 | GER | 1940 | Lignano | 14.09.2011 |
| 438 |  | 70-74 | GER | 13.01.1924 | Zeven | 16.09.1995 |
| 439 |  | 70-74 | FIN | 28.03.1936 | Lahti | 28.07.2009 |
| 440 |  | 70-74 | FIN | 30.04.1939 | Lahti | 28.07.2009 |
| 441 |  | 70-74 | FRA | 10.09.1920 | Turku | 19.07.1991 |
| 442 |  | 70-74 | HUN | 1917 | Eugene | 28.07.1989 |
| 443 | Multiple | 70-74 | USA | 02.10.1925 | Neosho | 20.09.1997 |
| 444 |  | 70-74 | GBR | 20.09.1935 | Oxford | 07.07.2007 |
| 445 |  | 70-74 | GBR | 12.01.1935 | San Sebastian | 23.08.2005 |
| 446 |  | 70-74 | RUS | 16.07.1940 | Sacramento | 07.07.2011 |
